# A Novel Mutation in the Maternally Imprinted *PEG3* Domain Results in a Loss of *MIMT1* Expression and Causes Abortions and Stillbirths in Cattle (*Bos taurus*)

**DOI:** 10.1371/journal.pone.0015116

**Published:** 2010-11-30

**Authors:** Krzysztof Flisikowski, Heli Venhoranta, Joanna Nowacka-Woszuk, Stephanie D. McKay, Antti Flyckt, Juhani Taponen, Robert Schnabel, Hermann Schwarzenbacher, Izabela Szczerbal, Hannes Lohi, Ruedi Fries, Jeremy F. Taylor, Marek Switonski, Magnus Andersson

**Affiliations:** 1 Lehrstuhl für Tierzucht, Technische Universitaet Munchen, Freising, Germany; 2 Department of Production Animal Medicine, University of Helsinki, Saarentaus, Finland; 3 Department of Genetics and Animal Breeding, University of Life Sciences, Poznan, Poland; 4 Division of Animal Sciences, University of Missouri, Columbia, Missouri, United States of America; 5 ZuchtData, EDV-Dienstleistungen GmbH, Wien, Austria; 6 Program in Molecular Medicine, Veterinary Biosciences, Department of Medical Genetics, Folkhälsan Institute of Genetics, Biomedicum Helsinki, University of Helsinki, Helsinki, Finland; King's College London, United Kingdom

## Abstract

Congenital malformations resulting in late abortions and stillbirths affect the economic wellbeing of producers and the welfare of cattle in breeding programs. An extremely high incidence of stillbirths of “half-sized” calves of normal karyotype and uninflated lungs was diagnosed in the progeny of the Finnish Ayrshire (*Bos taurus*) bull - YN51. No other visible anatomical abnormalities were apparent in the stillborn calves. We herein describe the positional identification of a 110 kb microdeletion in the maternally imprinted *PEG3* domain that results in a loss of paternal *MIMT1* expression and causes late term abortion and stillbirth in cattle. Using the BovineSNP50 BeadChip we performed a genome-wide half-sib linkage analysis that identified a 13.3 Mb associated region on BTA18 containing the maternally imprinted *PEG3* domain. Within this cluster we found a 110 kb microdeletion that removes a part of the non-protein coding MER1 repeat containing imprinted transcript 1 gene (*MIMT1*). To confirm the elimination of gene expression in calves inheriting this deletion, we examined the mRNA levels of the three maternally imprinted genes within the *PEG3* domain, in brain and cotyledon tissue collected from eight fetuses sired by the proband. None of the fetuses that inherited the microdeletion expressed *MIMT1* in either tissue. The mutation, when inherited from the sire, is semi-lethal for his progeny with an observed mortality rate of 85%. The survival of 15% is presumably due to the incomplete silencing of maternally inherited MIMT1 alleles. We designed a PCR-based assay to confirm the existence of the microdeletion in the *MIMT1* region that can be used to assist cattle breeders in preventing the stillbirths.

## Introduction

Artificial insemination has been used extensively in cattle breeding for over 50 years. An unfavorable side-effect of this technique is the risk of widely disseminating mutations that cause hereditary disorders. It is therefore very important from the perspective of population fitness to identify carriers of chromosome [1] or gene [2] mutations.

Fetal and placental development in mammals are both affected by imprinted genes for which either the paternally- or maternally-inherited allele has become epigenetically inactivated, leading to monoallelic expression. Approximately one half of the fetuses can be adversely affected when a male transmits a defective fully penetrant imprinted gene silenced on the maternal allele. More than 150 mammalian genes are known to be imprinted and these tend to be clustered in discrete chromosomal regions termed imprinted domains [3]. In mammalian cloning experiments the loss of the epigenetic control of imprinted genes has led to low success rates and the birth of animals with health problems [4],[5]. The pattern of inheritance of defects within imprinted genes can lead to significant problems for breeding programs. Defects in maternally imprinted genes will be transmitted silently from females to their progeny, while phenotypically normal males will transmit these mutations to their progeny as though they were heterozygous for a dominant mutation. If used in an artificial insemination program such males could produce hundreds or even thousands of progeny before they are detected as transmitting a genetic defect.

Very few mutations in imprinted regions are known that lead to disease phenotypes. However, one of the best characterized human maternally imprinted diseases is Prader-Willi syndrome [6],[7], which is usually caused by microdeletions within a maternally imprinted domain [6]. A well studied but incompletely understood imprinted phenotype in sheep is the point mutation in the maternally imprinted Callipyge locus [8],[9], which is expressed only in heterozygous genotypes when the Callipyge allele is inherited from the male parent.

The imprinted *PEG3* domain has been studied in mice, humans and cattle [10], [11],[12]. When *Peg3* was disrupted in mice, heterozygous progeny that inherited the null allele from their male parent were smaller, but otherwise phenotypically normal [13]. However, females harboring the paternally transmitted mutant allele had reduced fertility, milk production and aberrant maternal behavior. To our knowledge there has been no report of any mutation in the *PEG3* domain that has been associated with disease in humans. In particular, there has been no earlier report of mutations within the imprinted *PEG3* domain that cause increased rates of abortion and stillbirth in mammals. In one previous report a maternal duplication of the *Peg3*-region in mice was associated with neonatal lethality [14].

We describe a novel mutation in the *PEG3* domain that caused stillbirth and late abortion in 42.6% of all offspring in progeny that inherited the mutation from a carrier Finnish Ayrshire (*Bos taurus*) bull - YN51 whose semen was used to artificially inseminate more than 1,900 females.

## Results

### Presentation of the first bull used in artificial insemination with the mutation

**Figure 1:**
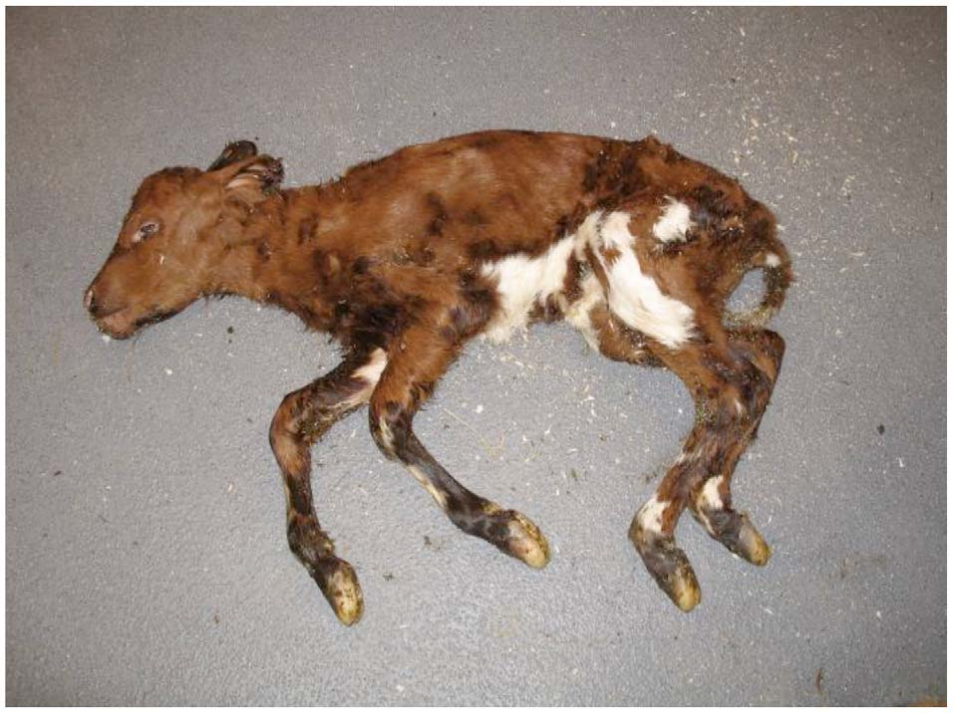
A dead small calf sired by the proband bull. The only visible anatomical difference between health and stillborn calves is the size. The stillborn calves are half-sized.

The semen of YN51 was commercially used in 2006 and 2007 to artificially inseminate 1,900 Finnish Ayrshire heifers and cows. Farmers began to report late gestation abortions and the stillbirth of small calves sired by this bull one year after the initial inseminations. Field data collected by the Artificial Insemination (AI) Cooperative indicated that 42.6% of the late pregnancies attributed to YN51 ended in stillbirths or abortions. In total, 318 late abortions and stillbirths were registered for offspring of YN51. The corresponding average percentage of late abortions and stillbirths for AI bulls of the Ayrshire breed is 5%. These results were unique and unacceptable, so a decision was made by the AI Cooperative to solve the etiology of the high proportion of abortions and stillbirths among the progeny of this bull. The stillborn calves were approximately 20 kg at birth or 50% the average normal birth weight for the breed, and the lungs were not inflated in the dead calves (Figure 1). No other visible abnormalities were detected in four stillborn calves subjected to necropsy. The stillbirth rate was normal (4%, 133 calvings) when the daughters of the proband calved. When YN51 was mated to Holstein females, 10 pregnancies resulted in 3 live born calves and 7 that were either stillborn, or aborted after at least 7 months of gestation. These results indicated that a phenotypically normal bull, YN51, was transmitting a lethal allele to approximately 50% of his purebred and crossbred progeny and that phenotypically normal daughters failed to transmit the allele to their progeny.

### Hypothesis for the genetic model

Based on extensive pedigree data representing thousands of bulls used in AI and millions of calvings, the extremely high incidence of the dead calf phenotype among the offspring of YN51 indicated that this bull possessed a unique mutation for which only a few modes of inheritance were possible. The most probable genetic models included either a mutation within a maternally imprinted domain or a chromosomal structural rearrangement. All of the studied semen parameters (e.g., motility) and early fertility parameters were within normal ranges and the bull had a normal karyotype (60, XY) with no abnormality detected by conventional staining and G-banding. Observed synaptonemal complexes under both electron and light microscopes revealed no abnormal pairing configurations indicative of a chromosomal rearrangement in any of the studied primary spermatocytes.

### Whole Genome Linkage Mapping

**Figure 2:**
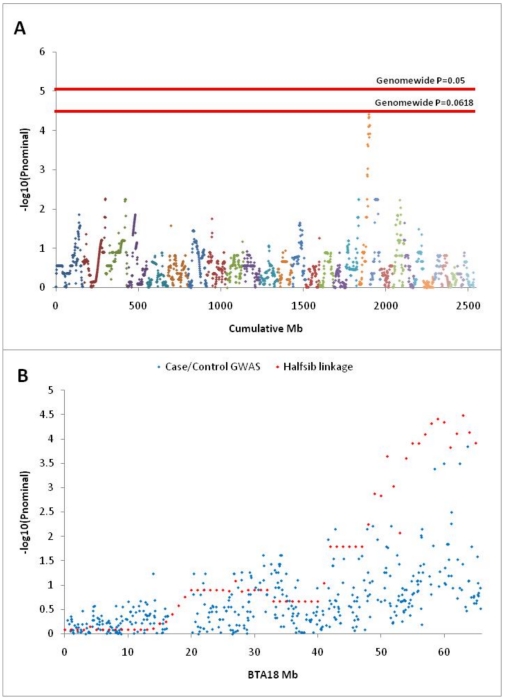
Genomic localization of the mutation responsible for early calf mortality among the progeny of Finnish Ayrshire bull -YN51. **A.** Genomewide half-sib linkage analysis performed with GridQTL for 15,631 autosomal loci for which YN51 was heterozygous. Plot resolution is 1 cM (assumed equivalent to 1 Mb) and phenotypes of 1 were assigned to each of the 5 affected calves and 0 to each of the 13 unaffected calves. The analysis localizes the casual mutation to BTA18 at genome-wide significance level of P = 0.0618. **B.** Linkage analysis and allele frequency association analysis performed for individual BTA18 SNPs localizes the mutation to the distal end of the chromosome.

Under the assumption that the causal mutation is maternally silenced and paternally expressed, the progeny of YN51 contain only linkage information concerning the genomic location of the disease locus. This is because only the alleles inherited from YN51 produce phenotypic effects in the progeny and thus no linkage disequilibrium information concerning the location of the disease locus can be extracted from the maternally inherited alleles. Accordingly, we performed a genome-wide linkage analysis using GridQTL [15] on five dead calves (A1–A5; carriers assigned a phenotype of “1”, Figure 2A) and 13 live calves (B11–B14, B16–B21, B24–B26; controls assigned a phenotype of “0”, Figure 2A) under a half-sib model using 15,631 single nucleotide polymorphisms from the Illumina BovineSNP50 assay for which YN51 was heterozygous. The analysis revealed the disease locus to be at the distal end of BTA18 (Figure 2B; P_genomewide_ = 0.062, N = 10,000 permutations).

To collect tissues for use in gene expression studies, eight additional cows, which for economic reasons had been scheduled for slaughter within 6 months, were inseminated with semen from YN51. At slaughter the placentas and fetuses were harvested and the tissues were immediately preserved in liquid nitrogen. The 8 fetuses (C1–C8) were also genotyped with the BovineSNP50 assay but no phenotypes could be assigned to the harvested calves.

**Figure 3:**
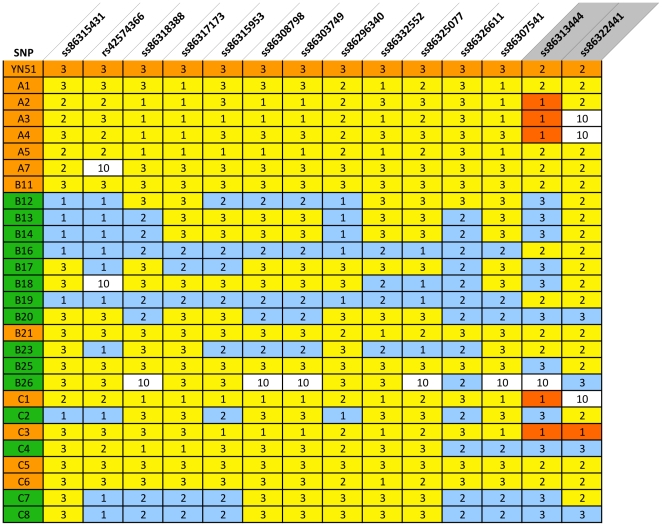
SNPs from the Illumina BovineSNP50 BeadChip that span the region harboring the disease locus. Genotypes are scored as 1 = *AA*, 2 = *BB*, 3 = *AB* and 10 =  missing. Upper orange row represent the SNP genotypes scored in the proband (YN51). All others rows represent half-sib progeny of YN51. Animal IDs in orange indicate animals that inherited the lethal haplotype. Animal IDs in green indicate animals that inherited the normal haplotype. Red fields indicate animals that inherited null alleles. C4 has a recombination, and has the normal haplotype from SNP ss8632661-onwards. Blue fields indicate genotypes not fitting with the mutation-associated haplotype with a deletion. A1–A7 were stillborn calves. B11–B26 represents live calves. C1–C8 represents fetuses. (B11 and B21 represent the two animals that survived despite inheriting the lethal haplotype).

**Figure 4:**
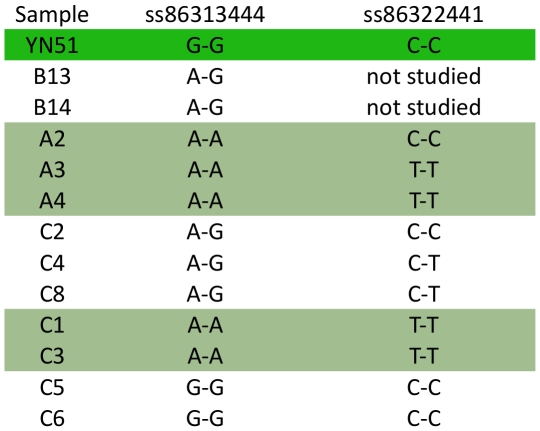
Genotypes for two consecutive SNPs in the associated region on BTA18. The genotypes for the SNPs ss86313444 at the position 64382789 bp and ss86322441 at 64411801 bp were determined by sequencing of PCR amplicons. YN51 is highlighted in green and progeny highlighted in light green inherited null alleles at these SNPs. Heterozygote animals do not have the deletion.

A haplotype analysis was performed with genotypes for all 40 animals including the sire of YN51, 18 calves, 8 fetuses and dams of 13 of the calves, and revealed that all five carriers had inherited the same lethal haplotype from YN51 across a 13.3 Mb region from 51446347 bp to 64727406 bp while only two of the 13 controls had inherited this haplotype (Table S1). Additionally, at least 4 of the fetuses (C1, C4–C6; and possibly also C3) possessed the lethal haplotype across this region (Table S1); additional SNP information is given in Table S2. Genotypes for two contiguous SNPs, ss86313444 at 64466896 bp and ss86322441 at 64495996 bp, demonstrated at least one Mendelian misinheritance between YN51 and his progeny (Figure 3). However, once haplotypes had been constructed for the region the genotypes for all 26 calves and fetuses and their genotyped parents were consistent with YN51 being heterozygous for a null allele at both loci. The presence of two null alleles at two flanking SNP loci separated by 29.01 kb strongly suggested the presence of a large deletion on YN51's maternally inherited chromosome. The deletion theory was tested for SNPs ss86313444 and ss86322441 by sequencing the loci from YN51 and the progeny which produced misinheritances from the BovineSNP50 assay and the sequence-based genotypes confirmed the homozygous genotypes called from the BovineSNP50 assay (Figure 4). These results are consistent with the deletion hypothesis. This region harbors the maternally imprinted *PEG3* domain, spanning 500 kb from 64.1 Mb to 64.6 Mb.

### SNP screening in candidate gene

**Figure 5:**
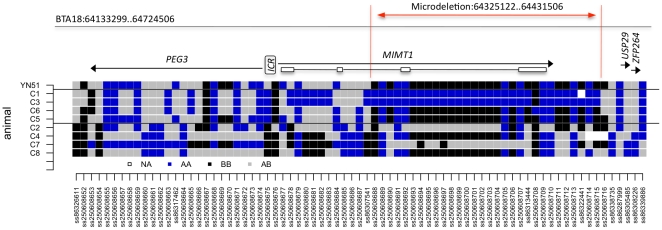
Genotypes for carrier and control bovine fetuses for SNPs within a region on BTA18 encompassing the *PEG3* locus. Fetuses C1–C8 are offspring of YN51. C1, C3, C5, C6 that inherited the lethal haplotype from YN51 and C2, C4, C7, C8 inherited the normal haplotype. ICR – imprinting control region. *MIMT1* is structured into 4 non-coding exons (open boxes). The genomic annotation of the region is based on the UMD3.1 assembly. SNPs with rs numbers are located on the BovineSNP50 assay, and short number IDs were assigned to the SNPs identified in this experiment. The microdeletion region is denoted by a red line.

The genomic organization of the bovine *PEG3* domain was annotated and edited using the Apollo sequence annotation viewer and based on the University of Maryland UMD3.1 release of the bovine genome assembly [16]. The *PEG3* domain is located in the distal 500 kb segment on BTA18 and includes the *PEG3*, *MIMT1*, and *USP29* genes [10]. The *MIMT1* is structured into 4 exons and was predicted to occur in a genomic region spanning about 80 kb between 64291075 and 64370359 bp (GenBank acc. nos. EF110915 and EF 110916). Applying a comparative sequencing approach, we constructed an additional dense panel of 79 SNPs in and around the region of interest (Figure 5, Table S2) to confirm the presence of a deletion and to evaluate the size of the deletion. The misinheritances and loss of heterozygosity (LOH) at 20 of the SNPs in carrier fetuses confirmed a microdeletion spanning approximately 110 kb between 64325122 and 64431506 bp that deletes exons 3 and 4 of *MIMT1* on one of the chromosomes in YN51. Due to the distance between SNP markers the estimated size of the deletion can range between 110 and 130 kb in the analyzed animals.

### The proposed inheritance model and independent confirmation of the deletion

Bull O71, the father of YN51, was also genotyped with the BovineSNP50 assay and was found to be heterozygous in the deletion area, and thereby deemed to be free of the deletion. This result was expected since O71 had 33 sons other than YN51 that were used as AI bulls, and none of the 33 half-siblings of YN51 had any problems with abortions or stillbirths. Unfortunately, only 4–5 hair-straws with root bulbs were available from O73, the mother of YN51. However, we succeeded in analyzing two SNPs in the deletion area and O73 tested heterozygous for one of the loci. Based on these results we conclude that O73 was probably deletion free and that the deletion was probably a *de novo* mutation in YN51.

**Figure 6:**
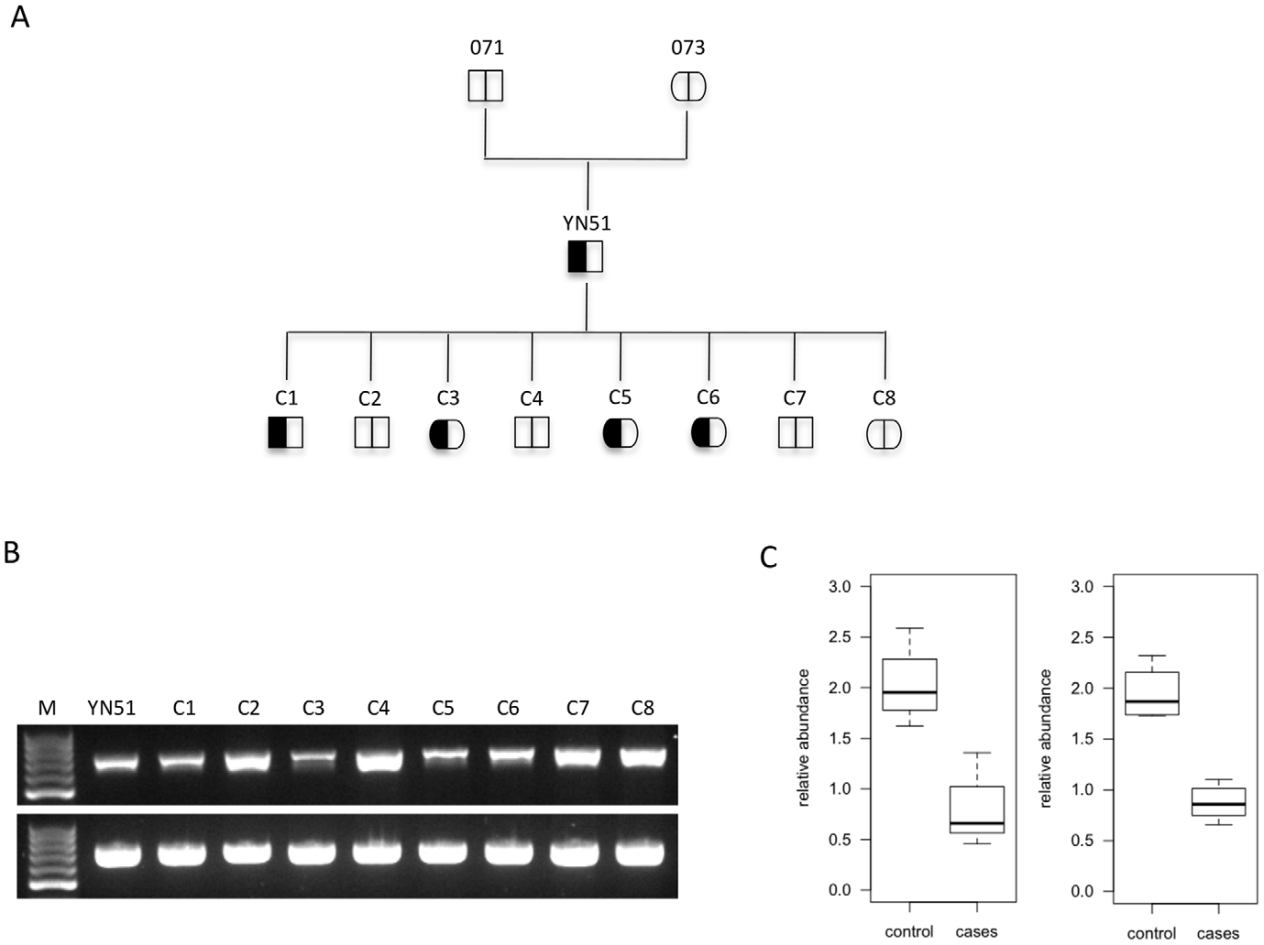
Loss of a *MIMT1* parental allele. **A.** Proposed (observed) inheritance model for the *MIMT1* microdeletion in the progeny of Finnish Ayrshire bull (*Bos taurus*). The deletion allele is denoted as a filled field, the normal allele as an open field. **B.** Agarose gel analyses of target and control PCR products demonstrating the amplification of either one or two genomic DNA copies. PCR product (737 bp) amplified with primer pair localized at the 3′end of *MIMT1* - upper picture. YN51, C1, C3, C5, C6 show PCR amplification from a single template chromosome; C2, C4, C7, C8 show PCR amplification from two template chromosomes. PCR (763 bp) of control amplification (lower picture) with primer pair localized in intron 2 of the *PEG3* gene. **C.** qPCR quantification of two different genomic regions within *MIMT1* locus calculated using the 2^∧(1+ (-ΔΔct))^ formula. Amplicon localized in exon 3 (left panel), and in 3′end of *MIMT1* (right panel). Genomic fragments on BTA26 with confirmed double-copy amplification, and BTA5 were used as control regions.

The second approach that we applied to study the presence of the deletion was to take the qPCR-based copy number detection approach. The amplification results from YN51, C1, C3, C5 and C6 putatively carrying the microdeletion were pooled and tested against the signals from animals C2, C4 (C4 is a recombinant for the normal haplotype in the region of the deletion, Table S1), C7 and C8, putatively carrying two template chromosomes. The results confirmed the presence of the microdeletion in the *MIMT1* genomic region in YN51, C1, C3, C5 and C6 (Figure 6).

### Candidate gene expression

**Figure 7:**
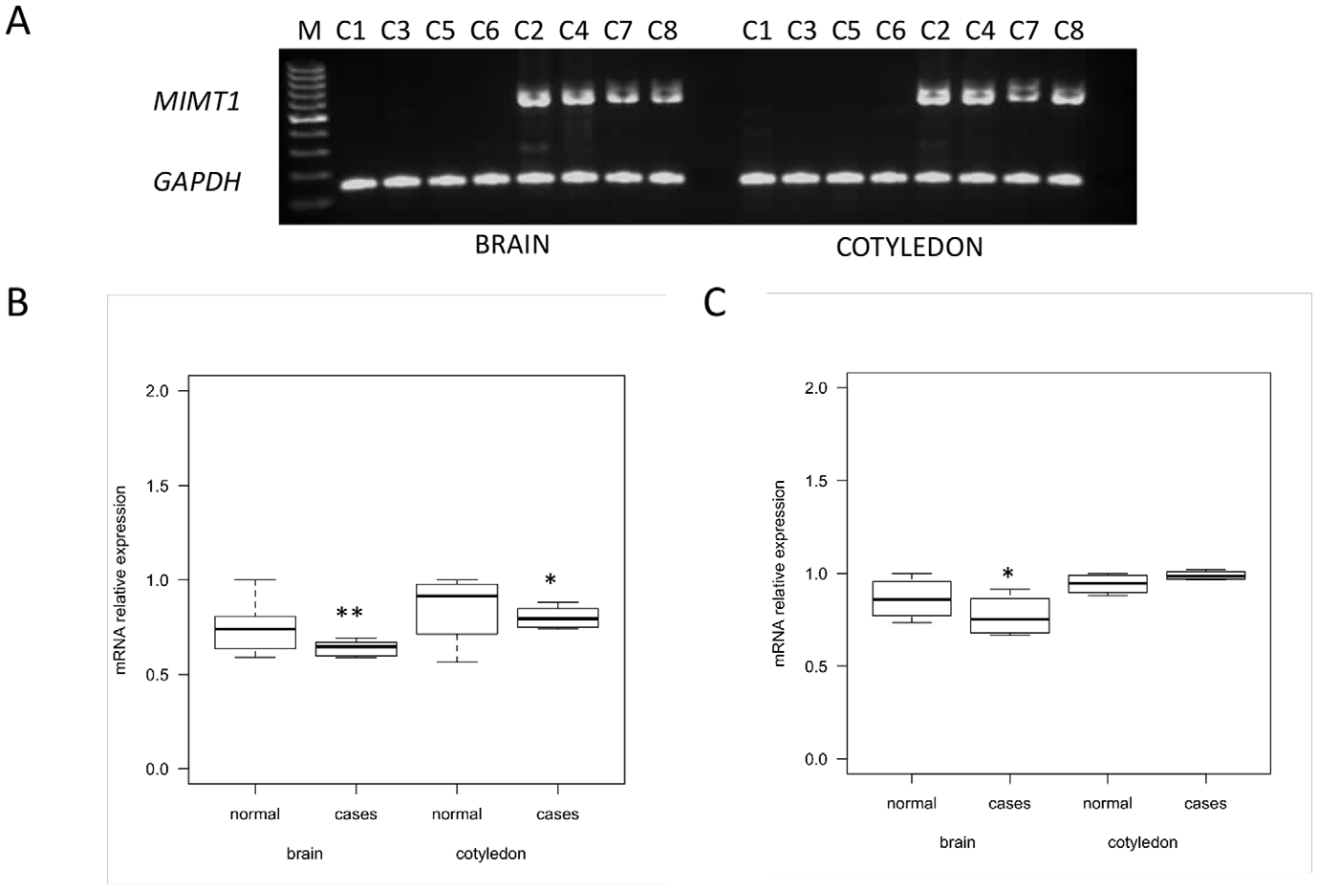
Expression analysis of the bovine *PEG3*, *MIMT1* and *USP29* genes. The fetal brain and cotyledon tissues were studied in four fetuses (C1, C3, C5, C6) that inherited the microdeletion chromosome from YN51 and four, which inherited the alternate normal chromosome (C2, C4, C7, C8) **A.** RT-PCR analysis of *MIMT1* gene. Due to the alternatively spliced exon 2 of *MIMT1* gene, two different-sized bands are visible in control fetuses. No product was detected in carrier fetuses. *GAPDH* RT-PCR was used for confirmation of reverse transcription efficiency. **B.** Box plot showing qPCR analysis of *PEG3* between control and case group. **C.** Box plot showing qPCR analysis of *USP29*. qPCR expression is shown as a relative level of mRNA calculated with the 2^∧(-ΔΔct)^ formula and using *GAPDH* as an internal control gene. pValue * <0.05, ** <0.01.

Based on the localization of the deletion, we expected *MIMT1* expression to be disrupted in progeny that inherited the deletion chromosome from YN51. Gene expression of *MIMT1*, *PEG3* and *USP29* was examined using quantitative real time PCR in fetal brain and cotyledon tissue from the fetuses C1–C8. The cotyledon is the placental structure on the fetal side (“*pars foetalis*”) and in normal fetuses these three genes should be expressed in both tissues [10]. Two different-sized alternatively spliced *MIMT1* transcripts, 644 and 737 bp in length, were detected in control fetuses, while in all four foetuses, which inherited the deletion haplotype from YN51 the expression was absent (Figure 7A). We sequenced the corresponding RT-PCR products and found that the nucleotide sequence of exon 2 is alternatively spliced, confirming previously published findings [10]. The expression of *PEG3* was lower in the fetus group, which inherited the deletion YN51 haplotype, but was detectable in all fetuses (Figure 7B). The expression of *USP29* in brain tissue was lower in the fetus group with the deletion haplotype, but was detectable in all fetuses (Figure 7C).

## Discussion

Here we describe a novel mutation in the maternally imprinted *PEG3* domain causing stillbirth and abortions in cattle. The mutation is a deletion of approximately 110 kb, starting in the *MIMT1* gene, including the 3′UTR, and that causes a loss of *MIMT1* expression in the brain and cotyledon in all carrier fetuses. Application of a high-resolution SNP panel to the DNA from these fetuses clearly demonstrated that the deletion had eliminated the 3′ end of the *MIMT1* gene that then abolished expression of the whole *MIMT1* transcript. This was confirmed in all four fetuses with the deletion haplotype, whereas the four fetuses with the normal haplotype expressed *MIMT1* in both brain and cotyledon tissue. The manner in which the *MIMT1* mutation affects the survival of fetuses is not clear. Since *PEG3* and *MIMT1* share a bidirectional promoter, one possibility is that *MIMT1* expression mediates transcriptional silencing of *PEG3* and/or *USP29* throughout bidirectional transcription. The mechanism of bidirectional transcription has not yet been fully explored, however its role in maintaining an open chromatin structure at promoters has been proposed [17]. This could not be reliably evaluated in this work because of the limited number of sampled fetuses. However, there appeared to be a lower expression of *PEG3* and *USP29* in the fetuses carrying the deletion than in the four fetuses without the mutation. Another suggested mechanism is the loss of imprinting (LOI) of *PEG3* and/or *USP29* mediated by silenced expression of the paternal *MIMT1* allele. A similar epigenetic mechanism has previously been described for *IGF2* and the *H19* gene cluster in mouse [18], [19]. Moreover, such assumption could not be verified in this study due to the lack of polymorphic coding SNPs within the genes of interest.

The mutation was associated with late fetal death and stillbirth in at least 42.6% of the offspring of YN51. This suggests that only 85% of the late fetuses with the mutation in the imprinted area die. This appears to be confirmed by the fact that two of the 13 control animals (15.4%) carried the deletion chromosome but were phenotypically normal. This is likely due to the incomplete silencing of maternally imprinted alleles, which is a common phenomenon for imprinted loci [20], [21], [22]. Crossbreeding with Holstein heifers and cows did not impact the survival rate of fetuses, which was consistent with the imprinting model of inheritance. However, the surviving female calves with the deletion should transmit the mutation to 50% of their offspring without any impact on fetal death, and bull calves inheriting this mutation from these dams could regenerate the problem if used extensively in AI. The identification of the causal mutation now allows the testing of YN51's descendants to eliminate this mutation from the Finnish Ayrshire population. The deletion appears to be a *de novo* mutation in YN51, but we cannot exclude YN51's mother as being a germ-line mosaic. The maternal transmission will be studied during the coming two years when blood samples will be collected in approximately 50–80 dairy herds in Finland.

This is the first report that demonstrates that a mutation, other than a disomy, in the imprinted *PEG3* domain causes a high rate of late abortions and stillbirths in cattle. Despite the fact that the biological role of *MIMT1* is unknown, our work demonstrates that a natural knockout results in prenatal lethality in mammals. Taken together with *Peg3* knockout work in mice, our findings suggest a regulatory mechanism affecting late prenatal developmental through *Peg3* expression.

## Materials and Methods

### Ethics statement

Procedures in living animals were limited to the collection of blood by jugular venipuncture or hairs pulled from the skin. Both procedures were conducted according to standard veterinary protocols and inflicted minimal, if any, pain to the animal. Finnish Governmental institutional committee approved all animal work, Decision no. STH051A.

### Animals

DNA was sampled from 40 animals including the proband, 18 calves, 8 fetuses and 13 dams. Limited DNA was available from the mother of YN51. Tissue samples for RNA studies were collected from fetuses C1–C8.

### DNA Extraction

Genomic DNA was isolated from EDTA-preserved hairs and tissue using the DNeasy Blood and Tissue kit (Qiagen Inc., Valencia, CA) following the manufacturer's protocol. DNA was eluted and diluted in MilliQ water, hair lysates were prepared for PCR from hair bulbs as previously described [23].

### Sperm analysis and early fertility records

Semen analysis for YN51 consisted of initial and post-thaw motility studies of each ejaculate intended for processing and finally used for artificial insemination. The sperm concentration and the total sperm content of each ejaculate were evaluated. A smear was made from two ejaculates for morphological examination of spermatozoa. The early fertility data were evaluated by the corrected non-return rate index (within 60 days of each insemination, which is a standard procedure for each AI-bull in Finland) for the inseminations.

### Lymphocyte culture and somatic chromosome analysis

Peripheral blood samples were collected from YN51 in heparinized tubes, and routine lymphocyte culture was established. The slides were Giemsa-stained and G-banded. International nomenclature of bovine chromosomes was followed [24].

### Synaptonemal complex analysis

Immediately after slaughter the testicles of YN51 were collected and transported to the laboratory. Spreading of the spermatocytes on 0.2 M sucrose droplets was performed as earlier described [25]. Two types of specimens were prepared for observation under electron and light microscopes. For electron microscope observation the sucrose droplets were placed on coated microscopic slides with 4% necoloidine amyl acetate solution. The spermatocytes were fixed in 4% paraformaldehyde, in 0.1 M sucrose. Staining with the use of 4% phosphotungstic acid (PTA) in ethanol, for the electron microscope, and 50% aqueous solution of silver nitrate, for the light microscope, was applied. The necoloidine film, with fixed and PTA stained spermatocytes, was removed from the microscopic slide on a water surface and then 100 square copper mesh was placed on the slide and it was picked up with parafilm. On slides for light microscopy several drops of silver nitrate were placed on the slide, which was then covered with a nylon mesh. The slides were stored in a humid chamber for 2–3 h at 60 C, rinsed with water and briefly counter-stained with 4% Giemsa solution. Microscopic observations were conducted under a Joel 1200 EX II electron microscope and a Nikon Eclipse 600 light microscope.

### Single-nucleotide polymorphism (SNP) analysis

A commercially available high-density single nucleotide polymorphism (SNP) assay (Illumina BovineSNP50 BeadChip, (Illumina, San Diego, CA, USA) [26] was used to genotype DNA at the University of Missouri, Columbia, USA. The DNA samples were collected from YN51 and 26 half-sib progeny sired by the bull. These half-sibs included: 5 stillborn calves, 14 live offspring and 8 fetuses (collected after the slaughter of their pregnant dams). In addition to these samples, the DNA of the mothers of 13 of the calves and of the father ofYN51 was also genotyped. In total, 40 animals were genotyped using the BovineSNP50 assay in this study. Genome-wide linkage analysis was performed using GridQTL [15] and custom software under an allele frequency association model and haplotypes for the associated region on BTA18 were constructed manually.

### Gene sequencing

Confirmation of SNP genotypes was performed using a comparative sequencing approach in 9 animals comprising YN51 and eight (C1–C8) half-sib progeny. PCR products were sequenced using a 3130xl Genetic Analyzer (Applied Biosystems). Briefly, the PCR (20 µl) contained 20 ng of genomic DNA, 1.5 mM MgCl_2_ in 1x PCR buffer, 0.5 µM of both, forward and reverse primer, 200 µM of each nucleotide, and 0.5 U of Taq DNA polymerase (Qiagen). The quality of the resulting PCR products was tested by 1.5% agarose gel electrophoresis. The PCR products were purified with 1 U of Exonuclase I (Fermentas), and 5 U of Shrimp Alkaline Phosphatase (Fermentas). The 10 µl of sequencing reaction mix was prepared using the forward and the reverse primer (0.5 µM), 1 µl of purified PCR-Product and BigDye Terminator v1.1 Sequencing Kit (Applied Biosystems). Sequencing products were then run on an ABI 3130xl automatic sequencer (Applied Biosystems). Multiple sequence traces from control and case animals were aligned and compared using the phredPhrap program from the Consed package (www.genome.washington.edu).

### Gene expression analysis

Gene expression analyses were performed for paternally expressed gene 3 (*PEG3*), MER1 repeat containing imprinted transcript 1 (*MIMT1*) and Ubiquitin specific peptidase 29 (*USP29*). The expression studies were performed on brain tissue and from cotyledons from the eight fetuses from dams slaughtered between days 41–157 of pregnancy (the normal gestation length in this *Bos taurus* breed is 280±10 days). The tissue samples were immediately frozen in liquid nitrogen and stored at −80°C for further analyses; primer sequences are given in Table S3.

Total RNA was extracted using Trizol (Invitrogen) according to the manufacturer's protocol with some modifications. After *DNase*I treatment (Fermentas), the RNA was spectrophotometrically quantified using a NanoDrop ND-1000 (PeqLab) and the RNA integrity was determined by 1% denaturing EtBr agarose gel electrophoresis. Complementary DNA (cDNA) was synthesized using the First Strand cDNA Synthesis Kit (Fermentas).

*MIMT1* mRNA abundance was measured by reverse transcriptase RT-PCR. PCR primers 7484/7485 were designed in exon 1 and exon 3 of the gene to amplify two products of length 737 bp and 644 bp (without exon 2 sequence), respectively. PCR was performed in 20 µl reaction volumes containing diluted first-strand cDNA equivalent to 50 ng of input RNA. PCR products were loaded on 1.5% agarose gels to compare band intensities and sizes among individuals that inherited alternate chromosomes from YN51. *GAPDH* was amplified as an endogenous control gene.

mRNA transcript levels for *PEG3* and *USP29* were measured by quantitative PCR (qPCR) using Fast SybrGreen MasterMix (Applied Biosystems) and run on an ABI7500 (Applied Biosystems). All cDNA samples were assayed in triplicate. Primer specificity and capture temperature were determined by melt curve analysis. The relative expression levels were normalized to the endogenous control *GAPDH* gene.

### qPCR-based copy number detection

PCR-based assays were used to estimate the relative copy number of DNA template when amplified from target and control regions; primer sequences are given in Table S3. Copy numbers were determined semi-quantitatively by standard PCR and by quantitative real-time PCR using SybrGreen detection chemistry (Applied Biosystems), in reaction mixes containing 20 ng of genomic DNA.

The PCR amplification products (737 bp) were produced with primer pair 7678/7679 and loaded on 1.5% agarose gels. The band intensities were visualized by GelDoc System (Intas, Germany) and compared among individuals that inherited alternate chromosomes from YN51. PCR amplification of a 763 bp region from intron 2 of *PEG3* with primer pair 7562/7563 was used as a control region. qPCR was performed for two different genomic regions of the *MIMT1* gene; product 1 (129 bp) amplified with primer pair 7482/7485 is localized in exon 3, and product 2 (177 bp) amplified with primer pair 8319/7679 is at the 3′end of the *MIMT1* gene. For each sample, the same concentration of the genomic DNA was used, and the amplification rate was highly correlated with the copy numbers at the deletion region. Triplicate cycle thresholds (CTs) were averaged and then normalized to the products produced from the control primer pairs. Assuming amplification of two template copies of genomic DNA from the control region, the relative copy number for each target region was calculated as 2^∧(1+ (-ΔΔct))^. The corrected CTs from all tested samples tended to form two discrete clusters. Genomic fragments on BTA26 and on BTA5 [27] were used as control regions.

## Supporting Information

Table S1 Inferred paternally inherited haplotypes for the distal 20.3 Mb of BTA18 for 5 affected calves (A1-A5 shaded in red), 13 normal calves (B11-B14, B16-B21, B24-B26, shaded green) and 8 preterm calves harvested at slaughter of the dam and for which phenotype was not known (C1-C8). Haplotypes were inferred for loci heterozygous at YN51 (including 3 loci for which YN51 was genotyped to be homozygous but appeared to be segregating for null alleles) using BovineSNP50 genotypes produced on the 26 calves, their sire YN51, the sire of YN51 (paternal grandsire of the calves) and the dams of A1, A3–A5, B11–B14, B21, and B24–B26. Red shaded haplotype blocks represent components of the haplotype that YN51 inherited from his dam harboring maternally silenced alleles, and the green shaded haplotype blocks represent components of the haplotype that YN51 inherited from his sire harboring paternally expressed alleles. Locus coordinates in italics and bold indicate the region of the chromosome that is most parsimonious with the hypothesis of a paternally expressed lethal mutation. No more than two of the normal calves (B11 and B21) inherited the “lethal” paternal haplotype for this region. The positions for Illumina BovineSNP50 BeadChip SNPs are based on the Btau4.0 assembly.
(XLS)

Table S2 SNP information.
(XLS)

Table S3 List of Primers.
(XLS)
